# Outcomes of cataract surgery in Microophthalmia

**DOI:** 10.12669/pjms.346.14622

**Published:** 2018

**Authors:** Saima Majid, Asim Ateeq, Sadia Bukari, Munawar Hussain

**Affiliations:** 1 *Dr. Saima Majid, FCPS. Department of Ophthalmology, Isra Postgraduate institute of Ophthalmology, Karachi, Pakistan*; 2 *Dr. Asim Ateeq, FCPS. Department of Ophthalmology, Isra Postgraduate institute of Ophthalmology, Karachi, Pakistan*; 3 *Dr. Sadia Bukhari, FCPS. Department of Ophthalmology, Isra Postgraduate institute of Ophthalmology, Karachi, Pakistan*; 4 *Dr. Munawar Hussain, M.S.Ophth. Department of Ophthalmology, Isra Postgraduate institute of Ophthalmology, Karachi, Pakistan*

**Keywords:** Micro ophthalmia (Mo), Congenital cataract (Cc), Intra ocular pressure (Iop), Posterior synachae (Po), Visual axis obscuration (Vao)

## Abstract

**Objective:**

To find out outcomes of cataract surgery with Microophthalmia in children less than two years

**Methods:**

This cross-sectional study was conducted at Al-Ibrahim Eye Hospital, Karachi from January 2016 to August 2017. It included thirty micro ophthalmic eyes of infants with visually significant cataract of age less than two years who had cataract surgery without intraocular lens implantation. Axial length of globe was 19 mm or less in all thirty eyes of seventeen infants. in which thirteen infants had bilateral cataract and four had unilateral cataract.

**Result:**

Thirty Simple Micro ophthalmic eyes from seventeen patients having visually significant congenital cataract were studied. Thirteen had bilateral cataract and four had unilateral cataract. Mean preoperative IOP was 9.0±1.2 mmHg and postoperative IOP after three months was 10.9±3.2 mmHg. Three patients had secondary capsular opacification 17.6%. Two patients had posterior synachae 11.8% after three months.

**Conclusion:**

The results suggest that cataract surgery in simple microophthalmia is safe procedure. Postoperative complications in this study were within acceptable limits. Long term study with intraocular lens is required to confirm our observation.

## INTRODUCTION

Congenital cataract is most common cause of treatable blindness in children.^1^ Visual system development depends in children if sharp, clear and focused images are formed on the retina of both eyes. Cataract hinders focused images on retina. So, Early detection, surgery and follow-up visits have significant roles in the restoration of a child’s vision in the case of congenital cataract with significant media opacity, otherwise amblyopia can be observed.^2^ Microphthalmia is defined as a globe with total axial length that is at least two standard deviations below the mean for age.^3^ Microphthalmia birth prevalence has been generally estimated to be 14 per 100,000 population.^4^

In infants it is essential to correct aphakia as soon as possible after removing cataract. One can implant an IOL after surgery but it is not that simple because at birth human lens is more spherical than in adults and has power of about 30D, which compensates for the shorter axial length of a baby’s eye.^5^ It is more problematic in simple micro ophthalmic eyes so after removing cataract, aphakik glasses are prescribed to avoid amblyopia.

If cataract is larger than 3 mm in diameter in the centre of pupil or unilateral cataract associated with strabismus, or bilateral cataract along with nystagmus, all are considered as visually significant.^6^ The assessment of visual acuity and extraocular motility and alignment in infants and young children presents innumerable challenges for the clinician.^7^ It is difficult to check in preverbal children who are unamenable for standard visual acuity testing, fixation behavior, fixation preference, and objection to occlusion.^8^ However, it is utmost important to work on every aspect.

Aim of this study was to preclude the development of expected stimulus-deprivation amblyopia because of cataract and avoid major postoperative complications which can warrant long-term vision surveillance. Children undergoing cataract surgery early in life like increased IOP, posterior synachae, visual obscuration i.e. thick posterior capsule.

## METHODS

This study was conducted at Al-Ibrahim Eye Hospital, Karachi from January 2016 to August 2017. Miroophthalmia is rare presentation and study was of short duration so due to long probability. Convenience sampling, study was done on thirty eyes. Seventeen patients aged under two years were enrolled, thirteen patients had bilateral cataract, four patients had unilateral cataract with micro ophthalmic eyes with ocular trauma, inflammation, tractional retinal detachment, choroidal coloboma, aniridia and with persistent fatal vasculature were not included in study.

Detailed history was taken from patient’s parent including prenatal and birth history. With the help of handheld slit lamp, corneal clarity, anterior chamber, pupil, lens and posterior chamber were examined. Pupil was dilated 2.5% phenylpherine and 0.5% tropicamide for seeing type, location and density of cataract.

Objective visual acuity assessment was impossible due to dens cataract, young age and poor cooperation of patients in both scenario, preoperatively and postoperatively. So vision was checked by CSM method. That is Central Non-central, Steady Unsteady and Maintained. Dilated fundus examination was done with 20 Diopter and indirect ophthalmoscope to rule out post segment abnormalities. For the patient with hazy fundus view, ultrasonography (B-Scan) was done and to determine axial length Ascan was done with the help of Nidek US-800-Echoscan.

On day of surgery pupil was dilated with 2.5% phenylpherine and 0.5% tropicamide. Using limbal approach a small entry in cornea was made by 2.8 mm Keratome Sodium Hydrate 1% (Healon) was injected in anterior chamber. Anterior continuous curvilinear capsularrhexis (CCC) was done by capsulotomy needle and lens was aspirated by simcoe cannula. Then under high magnification posterior capsulorrhexis was done by automated cutter and anterior vitrectomy was performed. Triamciloneacetonide was used to stain remaining vitreous and more vitrectomy was done if needed. Wound was closed by 10 – 10 Nylon suture and Dexamethasone was injected intracamerally.

Patient was called for follow up on 1^st^ day, 7^th^ day, one month and three month interval. Postoperatively, patient was prescribed topical steroids (betamethasone 0.1 %) and topical antibiotic (moxifloxacin 0.5 %) and 2hourly/day and 1 % homatropine twice a day for one week then tapered it according to progress. On every visit anterior segment examination was done to see cornea clarity, synaechae, post capsule opacification. Vision was checked on 7^th^ day 1^st^ month and 3^rd^ month visits. After one month and three months IOP was checked by Perkin’s tonometer under general anaesthesia. Cycloplegic refraction was done with Retinoscopy after one week of surgery and aphakik glasses (aspheric) were given to each eye who had bilateral or unilateral cataract surgery. In unilateral cases, part-time occlusion was advised. Retinoscopy was repeated after one month and three months of surgery. Parents of patient were counseled about corneal suture removal after one month and intraocular lens implantation at the age of two years.

## RESULTS

Thirty Micro ophthalmic eyes from seventeen patients having congenital cataract were studied. There were ten (58.8%) male and seven (41.2%) female. Thirteen had bilateral cataract and four had unilateral cataract. Mean age of the patient was 9.5±3.3 months with minimum of five and maximum of eighteen months. There was no complication during surgery. At the time of surgery mean axial length was 14.4±2.4millimeters. Mean preoperative IOP was 9.0±1.2 mmHg and postoperative IOP was 10.9±3.2 mmHg after three months with significant P-value 0.042. Postoperative IOP was raised only in five (29.4%) patients and treated pharmacologically. Three patients had secondary capsular opacification (17.6%). Two patients had posterior synachae (11.8%).

## DISCUSSION

In this study we chose Micro ophthalmic eyes for cataract surgery. Generally, axial length of newborn’s eyeball is about 16 millimeters in diameter. In an infant, the eye grows marginally to a length of approximately 19½ millimeters and it progressively grow to the length of about 24-25 millimeters.^9^ An understanding of the comprehensive anatomy along with profound preoperative assessment will help individualize each case and empower the surgeon to better prepare to avoid obstacles that could be encountered during cataract surgery.^10^ Simple micro ophthalmia, means that their axial length was small but otherwise globe was normal.^11^ In present study, thirteen patients had bilateral congenital cataracts and four had unilateral cataracts without IOL implantation. One study suggests that in micro ophthalmic eyes of infants, primary IOL implantation is controversial due to unsubtle technical difficulties of implanting an IOL of adult size in these small eyes and also they got the small-diameter capsular bag in comparability of the IOL diameter, and post-operative complications like higher rate of Visual Axis Obscuration and refractive changes.^12^ Another study excluded microphthalmos (64% of respondents) for primary IOL implantation.^13^

Development of Secondary glaucoma after pediatric cataract surgery is an important postoperative complication. It is very difficult to diagnose and treat aphakic glaucoma because these children can remain asymptomatic, inspite of their high IOP.^14^ In this study intraocular pressure was taken preoperatively and postoperatively under general anesthesia, mean Pre Operative IOP was 9.0±1.2 mmHg and postoperative IOP after three months was 10.9±3.2 mmHg (Table-I). Postoperative IOP was raised in 5 (29.4%) patients where as one previous study by Shrikant P noted glaucoma in 13.5% patients.^12^ Mean age at time of surgery was 9.5±3.3 months, In one study it was quoted that patients at very younger age of 4.5 months or less developed glaucoma, irrespective of the implantation of IOL.^15^

**Table-I:** Pre and Postoperative IOP

Pre op baseline IOP	4th week IOP	P-value
9.0±1.2	10.5±2.9	0.063
4th week IOP	IOP at 3rd month	
10.5±2.9	10.9±3.2	0.083
Pre op baseline IOP	IOP at 3rd month	
9.0±1.2	10.9±3.2	0.042

Another study reports that incidence of aphakic glaucoma, even with modern surgical techniques should be considered in all patients who had pediatric cataract extraction. It enrolled 113 patients and Postoperative glaucoma was seen in 9.7% of eyes of children and all needed antiglaucoma medications and three of them (31.4%), needed surgical intervention.^16^ In our study seventeen patients were enrolled and five of them developed glaucoma and it was treated medically with combination of dorzalamide and timlol. None of the patient required glaucoma surgery.

In this study after removing cataract, patients were left aphakik and later glasses were prescribed after cycloplegic refraction with the help of retinoscopy. In another study, higher rate of complications were noted with Intraocular lens implantation in infants and further surgery was required during the first postoperative year than those infants who underwent lensectomy/vitrectomy surgery without IOL implant.^17^ There are also less chances of glaucoma in aphakik patients than psudophakik patients.^16^,^18^

Inflammatory response in infants is very intense and the visual axis obscurity by fibrous membranes proliferating on the intact anterior vitreous surface could take place.^19^ All patients had meticulous anterior vitrectomy after cataract removal to avoid posterior capsular opacification. Primary posterior capsulectomy combined with an anterior vitrectomy can decrease but cannot eliminate the rate of secondary membrane.^20^ A comparison with previous study shows posterior synachie in (35.7%), and PCO in (16.7%). However, in this study posterior capsular Opacification was observed and result showed that out of seventeen patients, three had PCO 17.6%. Two patients 11.8% had posterior synachae. Previous study shows almost same results.^21^ PCO was seen in three patients which was later managed by secondary surgical capsulatomy after three months.

### Limitations of the study

We could not assess visual acuity preoperatively due to dense cataract and early age. There is a need for longer follow up of microphthamic eyes complications after secondary IOL implantation, for which we are keeping patients in our records.

## CONCLUSION

The present study suggest that cataract surgery in micro-ophthalmia is safe and successful option in smaller eyes and results in better vision after surgery. It has also fewer complications like glaucoma, post synachea and visual obscuration. Therefore, it is suggested that patients with even small eyes should opt for cataract surgery.
